# The Rax homeoprotein in Müller glial cells is required for homeostasis maintenance of the postnatal mouse retina

**DOI:** 10.1016/j.jbc.2023.105461

**Published:** 2023-11-15

**Authors:** Takuya Yoshimoto, Taro Chaya, Leah R. Varner, Makoto Ando, Toshinori Tsujii, Daisuke Motooka, Kazuhiro Kimura, Takahisa Furukawa

**Affiliations:** 1 Laboratory for Molecular and Developmental Biology, Institute for Protein Research, Osaka University, Suita, Osaka, Japan; 2 Department of Ophthalmology, Yamaguchi University Graduate School of Medicine, Yamaguchi University, Ube, Yamaguchi, Japan; 3 Genome Information Research Center, Research Institute for Microbial Diseases, Osaka University, Osaka, Japan

**Keywords:** retina, Müller glial cell, inflammation, homeostasis, transcription factor

## Abstract

Müller glial cells, which are the most predominant glial subtype in the retina, play multiple important roles, including the maintenance of structural integrity, homeostasis, and physiological functions of the retina. We have previously found that the Rax homeoprotein is expressed in postnatal and mature Müller glial cells in the mouse retina. However, the function of *Rax* in postnatal and mature Müller glial cells remains to be elucidated. In the current study, we first investigated Rax function in retinal development using retroviral lineage analysis and found that Rax controls the specification of late-born retinal cell types, including Müller glial cells in the postnatal retina. We next generated *Rax* tamoxifen–induced conditional KO (*Rax* iCKO) mice, where *Rax* can be depleted in mTFP-labeled Müller glial cells upon tamoxifen treatment, by crossing *Rax*^*flox/flox*^ mice with *Rlbp1-CreERT2* mice, which we have produced. Immunohistochemical analysis showed a characteristic of reactive gliosis and enhanced gliosis of Müller glial cells in *Rax* iCKO retinas under normal and stress conditions, respectively. We performed RNA-seq analysis on mTFP-positive cells purified from the *Rax* iCKO retina and found significantly reduced expression of *suppressor of cytokinesignaling-3* (*Socs3*). Reporter gene assays showed that *Rax* directly transactivates the *Socs3* promoter. We observed decreased expression of Socs3 in Müller glial cells of *Rax* iCKO retinas by immunostaining. Taken together, the present results suggest that Rax suppresses inflammation in Müller glial cells by transactivating *Socs3*. This study sheds light on the transcriptional regulatory mechanisms underlying retinal Müller glial cell homeostasis.

The vertebrate retina is part of the central nervous system and takes charge of the first step of vision. The neural retina is composed of six types of neurons and a single type of glial cell, called Müller glial cells, all of which originate from a single population of multipotent progenitor cells (1, 2, 3). Müller glial cells are retina-specific and the most predominant glial subtypes in the retina (4, 5). The functional contribution of Müller glial cells to the retina is wide-ranging, and includes homeostasis, structure, and retinal circuit function. For example, Müller glial cells control the metabolism of various neurotransmitters (6, 7), prevent excitotoxic injury by the uptake of neurotransmitters from the synaptic cleft (8), remove degenerating cell debris (9), and control extracellular K^+^ levels, which contribute to modulating neurotransmission or maintaining homeostasis (10). The guidance and maintenance of cellular organization across the retina are supported by Müller glial cells (11, 12). Müller glial cell-mediated uptake of glutamate contributes to the separation of excitatory input into ON and OFF retinal ganglion cells (RGCs) (13).

On the other hand, Müller glial cells can also contribute to retinal regeneration. In fish and other lower vertebrates, Müller glial cells are involved in tissue maintenance and repair, as intrinsic retinal stem cells. In particular, when the zebrafish retina is damaged, Müller glial cells, called radial glia in zebrafish, operate as neural stem cells under both physiological and regenerative conditions and are able to differentiate into various neuronal cells, including photoreceptor cells and RGCs, to compensate and regenerate the damaged area (14, 15). While Müller glial cell-dependent retinal regeneration has not been considered to occur in mammals, recent studies have reported that Müller glial cells can cause functional production of new retinal neurons in adult mice by overexpressing a combination of proneural transcription factors, Ascl1 and Atoh1, in Mϋller glial cells with histone deacetylase inhibition (16, 17, 18). Another study showed that the loss of Nfia/b/x function induces Müller glia to proliferate and generate neurons in adult mice after injury (19).

Müller glial cell genesis is known to be regulated by multiple transcription factors, including Rax, Lhx2, Sox9, Nfia/b/x, and Hes family proteins, in association with the Notch pathway (20, 21, 22, 23). *Rax*, encoding a paired-type homeoprotein, is a crucial regulatory gene for early retinal development and is required for various processes of retinal development in vertebrates (24). In early mouse embryonic stages, *Rax* begins to be expressed in the optic vesicle and the putative diencephalon region, and then *Rax* expression shifts predominantly to the retina as development proceeds (25, 26). *Rax* is highly expressed in retinal progenitor cells (RPCs) and its expression gradually decreases as RPCs differentiate (25, 27). *Rax*-null mutant mouse embryos do not form optic vesicles and exhibit reduced brain structure (24, 26). Mutations in the *RAX* gene were reported in human microphthalmia, anophthalmia, and sclerocornea (28, 29). The role of *RAX* in human eye development is clearly supported by the linkage of symptoms in a patient with a truncating mutation and missense mutations, which are located in the region encoding the homeodomain of the RAX protein and reduces the DNA-binding ability of the resulting protein (28, 29). *Rax* paralog genes have been identified in a diverse range of vertebrate species (30, 31, 32). In the adult human retina, human *RAX2*/*QRX* is expressed in the outer nuclear layer (ONL) and inner nuclear layer, and functions synergistically with *CRX* and *NRL* to control photoreceptor gene expression (31). In chicks, two *Rax* genes (*cRax* and *cRaxL*/*cRax2*) have been identified (30). The *cRax2* gene is expressed in both RPCs and early developing photoreceptors, whereas *cRax* is predominantly expressed in retinal progenitors. The expression of a presumed dominant negative allele of the *cRax2* gene significantly reduces the expression level of cone photoreceptor genes, suggesting that *cRax2* regulates the differentiation of cone photoreceptor cells (30). Two *Rax* genes (*xRx* and *xRx-L*/*xRx2*) were identified in *Xenopus laevis* (26, 32), and three *Rax* genes (*zRx1* to *zRx3*) were isolated from zebrafish (26). It should be noted that the expression pattern in the retina of *zRx3* was more similar to that of frog and mouse *Rax* genes than to that of *zRx1* and *zRx2* genes (33). The genomes of monkeys, cows, and dogs also encode two *Rax* genes. From an evolutionary perspective, segmental duplication of the *Rax* locus occurred in an early common ancestor of jawed vertebrates, resulting in two *Rax* paralogs in jawed vertebrates, *Rax* and *Rax2* (34). The *Rax2* gene, on the other hand, is neither present in the mouse nor rat genomes (31). In mice, deletion of *Rax* at embryonic day 13.5 (E13.5) in photoreceptor precursors results in photoreceptor cell fate conversion to amacrine-like cells (35). In addition, a reduction in cone photoreceptor cells was observed when *Rax* was depleted in postnatal mouse retina (36). Thus, *Rax* plays important roles not only in the initial stage but also in the later developmental stages of the retina, including proliferation, cell fate determination, and photoreceptor maturation and maintenance.

We previously reported that in the mouse retina, after postnatal photoreceptor differentiation and maturation, *Rax* localization gradually shifts from photoreceptor cells to Müller glial cells (36). *Rax* overexpression in the postnatal day 0 (P0) mouse retina leads to an increase in the population of Müller glial cells (22). The gene expression profile of Müller glial cells is similar to that of RPCs (37, 38, 39). These studies imply that *Rax* possibly plays important roles in differentiated Müller glial cells at mouse postnatal stages; however, the function of *Rax* in postnatal retinal cell fate determination and in differentiated Müller glial cells is still unclear.

## Results

### *Rax* regulates the generation of late-born retinal cell types

**Figure 1:**
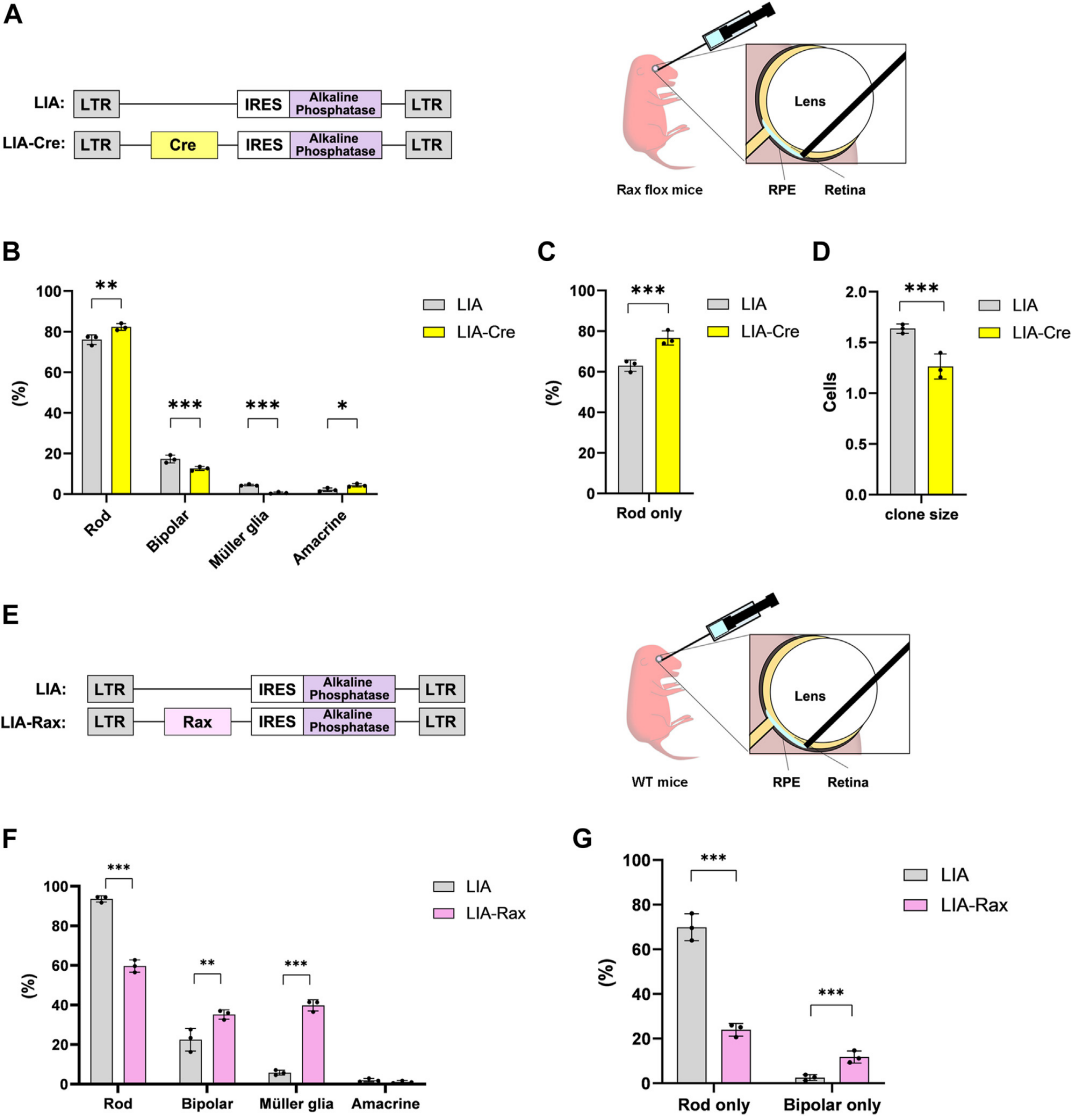
**The effect of *Rax* dosage on cell fate specification in postnatal retinal development.***A*, *left*: retroviral constructs used to express *Cre* and a marker gene, human placental alkaline phosphatase (AP), in the *Rax*^*flox/flox*^ mouse retina. *Right*: LIA or LIA-Cre virus was injected into the subretinal space of the P0 *Rax*^*flox/flox*^ mouse eye. The retrovirus-infected retinas were harvested at P28 and stained for AP. *B–D*, cell composition analysis of LIA- or LIA-Cre-retrovirus–infected clones. The percentage of rod photoreceptors (rod), bipolar cells, Müller glia, and amacrine cells out of the total number of cells infected with the LIA or LIA-Cre virus at P28 (*B*). The percentage of rod-only clones out of the total number of clones infected with the LIA or LIA-Cre virus at P28 (*C*). The average number of cells contained in each clone infected with LIA or LIA-Cre virus (*D*). *E*, *left*: retroviral constructs used to express *Rax* and *AP*, in the WT mouse retina. *Right*: LIA or LIA-Rax virus was injected into the subretinal space of the P0 WT mouse eye. The retrovirus-infected retinas were harvested at P28 and stained for AP. *F* and *G*, cell composition analysis of LIA- or LIA-Rax-retrovirus–infected clones. The percentage of rod photoreceptors (rod), bipolar cells, Müller glia, and amacrine cells out of the total number of cells infected with the LIA or LIA-Rax virus at P28 (*F*). The percentage of rod-only and Bipolar-only clones out of the total number of clones infected with the LIA or LIA-Rax virus at P28 (*G*) is shown. n = 3 retinas (1004 clones) for LIA and n = 3 retinas (774 clones) for LIA-Cre (*B* and *C*). n = 3 retinas (765 clones) for LIA, n = 3 retinas (613 clones) for LIA-Rax (*F* and *G*). Error bars, mean ± SD. Student’s *t* test (∗*p* < 0.05, ∗∗*p* < 0.01, ∗∗∗*p* < 0.001) was adopted.

To investigate the *Rax* function in retinal development, we performed retroviral lineage analysis using a murine replication-incompetent retrovirus, LIA, which only infects progenitor cells in the retina and encodes the human placental alkaline phosphatase (AP) gene (40). Retroviral vectors are infectious viruses that can introduce nonviral genes into mitotic cells *in vivo* or *in vitro* (41). The vectors useful for lineage analysis have been modified to be nonreplicable so that they cannot spread from one infected cell to another. However, these viruses are ideal for lineage analysis because they are faithfully passed on to all daughter cells of the first infected progenitor cell. We carried out a cell composition analysis on the descendants of *Rax*-overexpressed or KO progenitor cells by retrovirus infection at postnatal day 0 (P0). To examine the effects of *Rax* deficiency on retinal development, LIA or Cre coexpressing LIA (LIA-Cre) was injected into the subretinal space of P0 *Rax*^*flox/flox*^ mice (35), and retrovirus-infected retinas were harvested and analyzed at P28. The infected cells were labeled using AP histochemistry (Fig. 1*A*) (42). The LIA-Cre-infected retinas showed a significant increase in the percentage of rod photoreceptors and amacrine cells and a significant decrease in the percentage of late-born retinal cell types, bipolar and Müller glial cells, compared with the LIA-infected control retinas (Fig. 1*B*). In addition, the number of rod-only clones increased in the LIA-Cre-infected retinas (Fig. 1*C*). In contrast, we found that the average clone size, which indicates the number of descendant cells from a single progenitor cell, significantly decreased in LIA-Cre-infected retinas compared to that in LIA-infected retinas (Fig. 1*D*). To examine the effects of Rax overexpression on retinal development, LIA or LIA-Rax was injected into the subretinal space of P0 WT mice. Retrovirus-infected retinas were harvested and analyzed at P28 (Fig. 1*E*). The percentage of bipolar and Müller glial cells significantly increased, whereas the percentage of rod photoreceptors significantly decreased in LIA-Rax-infected retinas (Fig. 1*F*). We also observed a decrease in rod-only clones and an increase in bipolar cell-only clones in LIA-Rax-infected retinas (Fig. 1*G*). Many two-cell clones, especially bipolar cell-containing clones, are rod-bipolar cell mixture clones, suggesting a close relationship between the lineages of rods and bipolar cells (43). Therefore, counting and comparing rod-only and bipolar cell-only clones separately are useful for verifying the differentiation of rod and bipolar cells in detail. Consistent with a previous study (22), we could not quantify the clone size in the retinas of LIA-Rax-infected WT mice, since the Müller glia-like cells labeled with AP, which markedly increased with *Rax* overexpression, possessed extensive processes overlapping with neighboring cells expressing AP. These results suggest that Rax controls the specification of late-born retinal cell types, including Müller glial cells, in the postnatal retina.

### *Rax*-deficient Müller glial cells exhibit histological characteristics of reactive gliosis

We observed that the rates of decrease and increase in Müller glial cells by *Rax* deficiency and overexpression, respectively, were the highest among the retinal cell types examined (Fig. 1, *B* and *F*). In addition, we previously reported that Rax is highly expressed in Müller glial cells after retinogenesis is completed (36). These observations prompted us to investigate the *Rax* function in Müller glial cells. We first generated *Rlbp1-CreERT2* transgenic mice, in which *CreERT2* expression was driven by the *Rlbp1* 3.1 kb promoter (44), and crossed them with a reporter mouse line, *R26-CAG-LoxP-mTFP1* (Fig. S1*A*) (45). We injected tamoxifen into *Rlbp1-CreERT2*; *R26-CAG-LoxP-mTFP1* mice at P4, harvested their retinas at 1 month of age (1 M) (P4→1 M), and found that mTFP signals showed Müller glial cell morphology and overlapped with S100β (a Müller glial cell marker) signals (Fig. S1*B*). This observation showed that Cre recombinase activity can be induced in Müller glial cells by tamoxifen injection in *Rlbp1-CreERT2* mice. Cre-mediated recombination was observed in retinal pigment epithelium cells of *Rlbp1-CreERT2*; *R26-CAG-LoxP-mTFP1* mice treated with tamoxifen. However, in our previous study, we did not detect *Rax* expression in the retinal pigment epithelium cells of mice at the embryonic and postnatal stages (24, 36).

**Figure 2:**
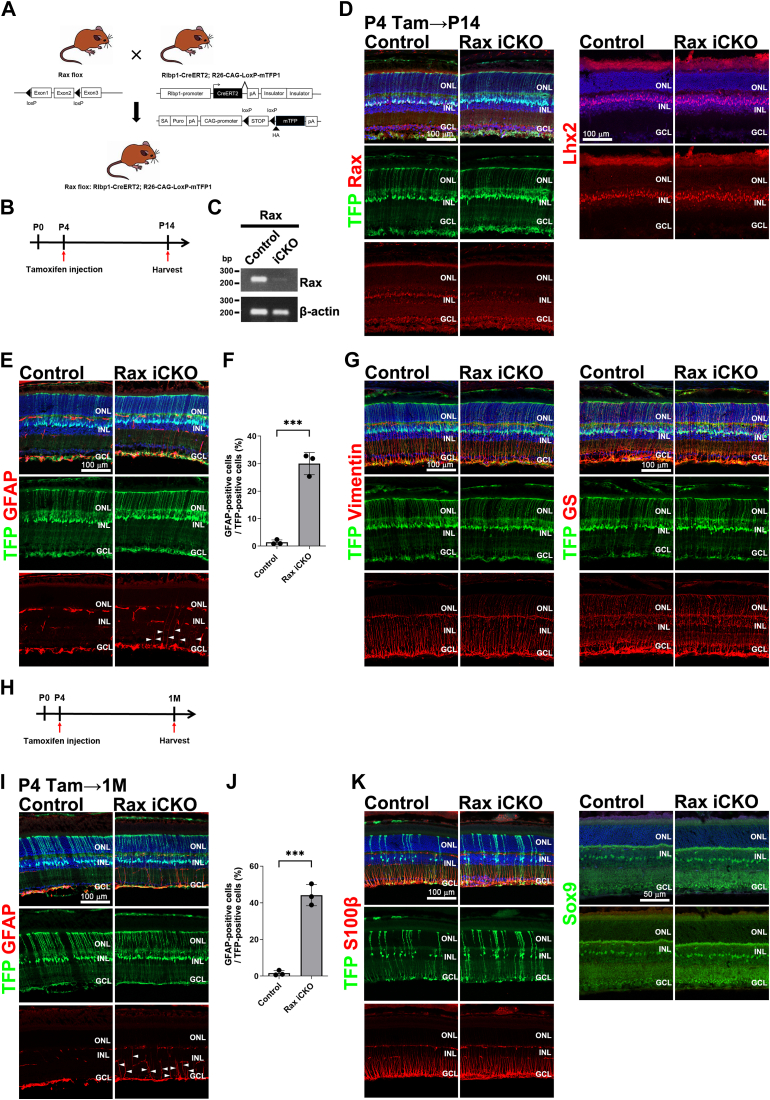
**Effects of *Rax* deficiency on Müller glial cells.***A*, schematic diagram of generation of *Rax* flox; *Rlbp1-CreERT2*; *R26-CAG-LoxP-mTFP1* mice. *Rax*^*flox/+*^; *Rlbp1-CreERT2*; *R26-CAG-LoxP-mTFP1* mice and *Rax*^*flox/flox*^; *Rlbp1-CreERT2*; *R26-CAG-LoxP-mTFP1* mice treated with tamoxifen were used as control and *Rax* iCKO mice, respectively, in the following experiments. *B*, schedule for tamoxifen injection and harvest of mice. Mice were injected with tamoxifen at P4 and harvested at P14 (P4→P14). *C*, RT-PCR analysis of the *Rax* transcript in mTFP-positive cells sorted by FACS from the control and *Rax* iCKO retinas (P4→P14). *β-actin* was used as a loading control. *D*, retinal sections from control and *Rax* iCKO mice (P4→P14) were immunostained with anti-Rax and anti-Lhx2 antibodies. Nuclei were stained with DAPI (*blue*). *E*, retinal sections from the control and *Rax* iCKO mice (P4→P14) were immunostained with an anti-GFAP antibody (a marker of gliosis in Müller glial cells). Nuclei were stained with DAPI (*blue*). GFAP signals increased in mTFP-positive cells of the *Rax* iCKO retinas (*arrowheads*). *F*, the percentage of GFAP-positive cells among mTFP-positive cells in *Rax* iCKO and control mouse retinas (P4→P14). ∗∗∗*p* < 0.001 (Student’s *t* test). n = 3 mice per group. *G*, retinal sections from the control and *Rax* iCKO mice (P4→P14) were immunostained with anti-Vimentin and anti-GS antibodies. Nuclei were stained with DAPI (*blue*). *H*, schedule for tamoxifen injection and harvest of mice. Mice were injected with tamoxifen at P4 and harvested at 1 M (P4→1 M). *I*, retinal sections from the control and *Rax* iCKO mice (P4→1 M) were immunostained using an anti-GFAP antibody. Nuclei were stained with DAPI (*blue*). GFAP signals increased in mTFP-positive cells of the *Rax* iCKO retinas (*arrowheads*). *J*, the percentage of GFAP-positive cells among mTFP-positive cells in *Rax* iCKO and control mouse retinas (P4→1 M). ∗∗∗*p* < 0.001 (Student’s *t* test). n = 3 mice per group. *K*, retinal sections from control and *Rax* iCKO mice (P4→1 M) were immunostained with anti-S100β (a Müller glial cell marker) and anti-Sox9 (a Müller glial cell marker) antibodies. Nuclei were stained with DAPI (*blue*). DAPI, 4',6-diamidino-2-phenylindole; FACS, fluorescence-activated cell sorting; GCL, ganglion cell layer; INL, inner nuclear layer; ONL, outer nuclear layer; Rax iCKO, *Rax* tamoxifen–induced conditional KO.

Next, we mated *Rlbp1-CreERT2*; *R26-CAG-LoxP-mTFP1* mice with *Rax*^*flox/flox*^ mice, injected tamoxifen into *Rax*^*flox/flox*^; *Rlbp1-CreERT2*; *R26-CAG-LoxP-mTFP1* mice at P4, and harvested their retinas at P14 (P4→P14) (Fig. 2, *A* and *B*). We observed that *Rax* expression was markedly decreased in mTFP-positive cells collected by fluorescence-activated cell sorting (FACS) from the retina of *Rax*^*flox/flox*^; *Rlbp1-CreERT2*; *R26-CAG-LoxP-mTFP1* mice treated with tamoxifen (*Rax* tamoxifen-induced conditional KO [*Rax* iCKO] mice) by RT-PCR analysis (Fig. 2*C* and S2*A*). We confirmed that Rax expression decreased in Müller glial cells by immunostaining using an anti-Rax antibody in *Rax* iCKO (P4→P14) retinas. We also performed immunostaining using an antibody against Lhx2, which has been suggested to be upstream of *Rax* (46) and found no substantial differences between the control and *Rax* iCKO (P4→P14) retinas (Fig. 2*D*). To examine the effects of *Rax* deficiency on Müller glial cells, we performed immunostaining using an anti-GFAP antibody (a marker for reactive gliosis in Müller glial cells) in *Rax* iCKO (P4→P14 and P4→1 M) retinas. We observed increased GFAP signals, a histological characteristic of reactive Müller cell gliosis, in mTFP-positive cells of *Rax* iCKO (P4→P14 and P4→1 M) retinas (Fig. 2, *E*, *F*, *I* and *J*). Next, we immunostained retinal sections using antibodies against Vimentin and glutamine synthetase (GS), other markers of reactive gliosis, and found that Vimentin signals increased in the ONL of the *Rax* iCKO (P4→P14) retinas compared with those of the control retinas (Fig. 2*G*). We also immunostained retinal sections using antibodies against S100β and Sox9 (Müller glial cell markers) and observed no obvious differences between the control and *Rax* iCKO (P4→1 M) retinas, suggesting that the number and morphology of Müller glial cells were unaffected by *Rax* deficiency (Fig. 2*K*). In addition, we immunostained for major cell types other than Müller glial cells in the *Rax* iCKO (P4→1 M) retina and found no substantial differences between the control and *Rax* iCKO retinas (Fig. S2, *B–H*). These results suggest that Müller glial cells undergo reactive gliosis because of *Rax* deficiency.

To examine the effects of *Rax* deficiency in Müller glial cells on retinal function, we measured electroretinograms (ERGs) of *Rax* iCKO (P4→1 M) mice under dark-adapted (scotopic) and light-adapted (photopic) conditions. We confirmed the efficiency of tamoxifen-induced recombination in the retina by immunostaining retinal sections after ERG analysis (Fig. S3*A*). Under scotopic conditions, the amplitude of a-waves and b-waves, originating mainly from the population activity of rod photoreceptor cells (a-waves) and rod bipolar cells (b-waves), was not significantly different between the control and *Rax* iCKO mice (Fig. S3, *B–D*). The implicit time of the scotopic ERG a-wave and that of the scotopic ERG b-wave, which is an indication of the speed of the transduction process from rod photoreceptors to rod bipolar cells, was also not significantly different between the control and *Rax* iCKO mice (Fig. S3, *E* and *F*). Similar to scotopic ERG, the amplitudes of photopic a-waves and b-waves, which mainly reflect the population activity of cone photoreceptor cells (a-waves) and cone ON bipolar cells (b-waves), in *Rax* iCKO mice were comparable to those in control mice (Fig. S3, *G–I*). The implicit time of the photopic ERG a-wave and that of the photopic ERG b-wave, which represents the speed of the transduction process from cone photoreceptor to cone ON bipolar cells, were also not significantly different between the control and *Rax* iCKO mice (Fig. S3, *J* and *K*). These results suggest that photoreceptor and ON bipolar cell functions are unaffected by *Rax* deficiency in Müller glial cells.

To investigate whether the increase in GFAP signals in Müller glial cells of the *Rax* iCKO retina is influenced by the timing of tamoxifen administration, we first injected tamoxifen into *Rax*^*flox/flox*^; *Rlbp1-CreERT2*; *R26-CAG-LoxP-mTFP1* mice at 1 M and harvested the retina at 2 M (1 M→2 M) (Fig. S4*A*). We performed immunostaining using the anti-GFAP antibody and did not observe an increase in GFAP signals in the mTFP-positive cells of the *Rax* iCKO (1 M→2 M) retina, in contrast to the observation in the *Rax* iCKO (P4→P14 and P4→1 M) retinas (Fig. S4*B*). We also immunostained retinal sections using the anti-Sox9 antibody and observed no obvious differences between the control and *Rax* iCKO (1 M→2 M) retinas (Fig. S4*B*). In addition, ERG analysis of the mice used for immunohistochemistry (Fig. S4*B*) showed no significant differences in the amplitudes and implicit times of scotopic and photopic ERG a- and b-waves between control and *Rax* iCKO (1 M→2 M) mice (Fig. S4, *C–L*). Next, we injected tamoxifen into *Rax*^*flox/flox*^; *Rlbp1-CreERT2*; *R26-CAG-LoxP-mTFP1* mice at P9 or P12 and harvested their retinas at 1 M (P9 or P12→1 M) (Fig. S4*M*). We did not observe a substantial increase in GFAP signals in the mTFP-positive cells of the *Rax* iCKO (P9 or P12→1 M) retinas (Fig. S4*N*). Given that retinogenesis is almost complete around P7 (47, 48, 49), these results suggest that the *Rax* deficiency in differentiating Müller glial cells can induce reactive gliosis in the retina.

### Reactive gliosis induced by retinal damage is enhanced by *Rax* deficiency in Müller glial cells

**Figure 3:**
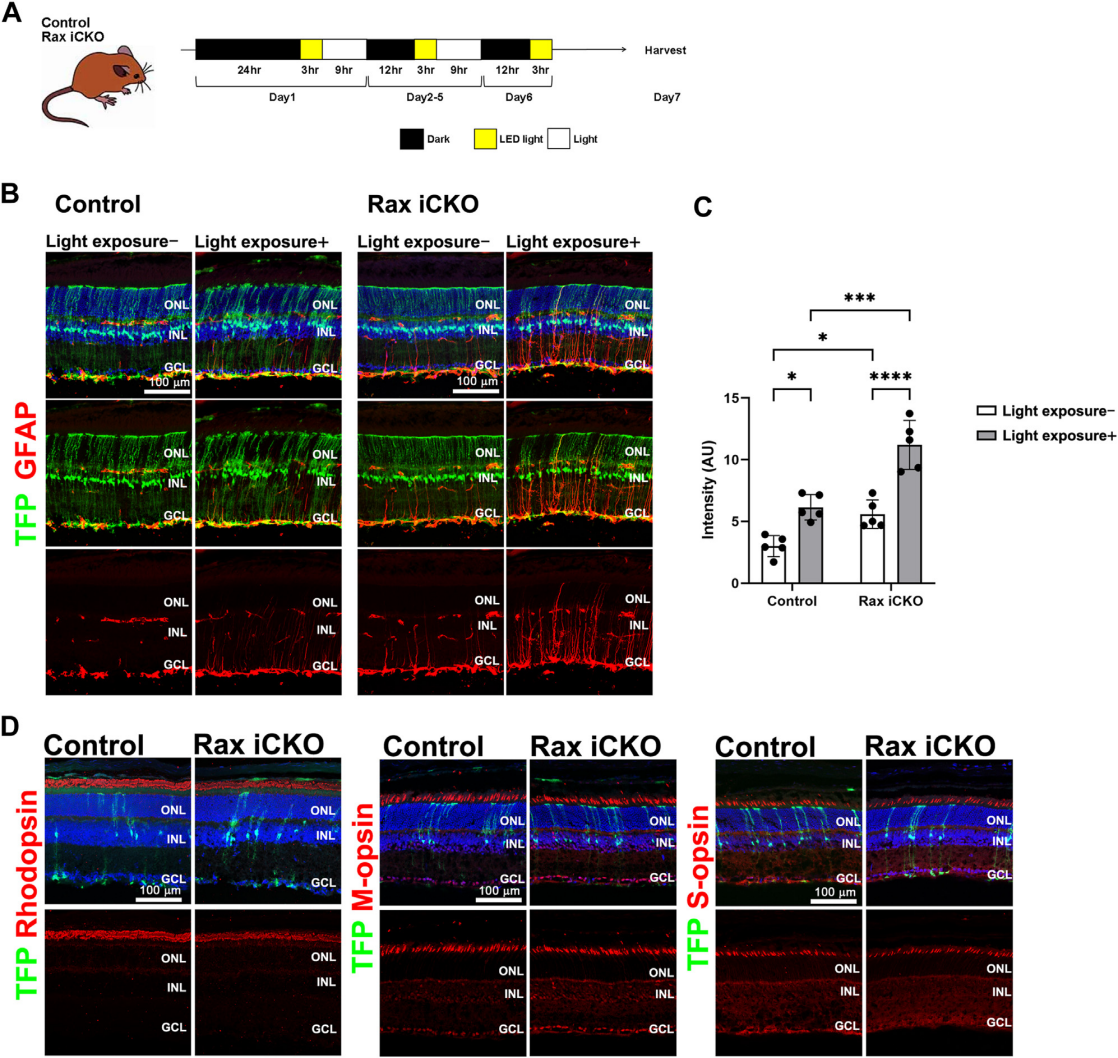
**Light-induced damage to *Rax* iCKO mice.***A*, experimental design for exposure of *Rax* iCKO and control mice to LED light. *B*, immunohistochemical analysis of retinas from *Rax* iCKO and control mice (P4→1 M) with or without light exposure using the anti-GFAP antibody. Nuclei were stained with 4',6-diamidino-2-phenylindole. *C*, the intensity of GFAP signals in the area between the ONL and GCL in the retinas of *Rax* iCKO and control mice (P4→1 M) with or without exposure to LED light. ∗*p* < 0.05, ∗∗∗ *p* < 0.001, and ∗∗∗∗*p* < 0.0001 (two-way ANOVA with Sidak’s multiple comparisons test). n = 5 mice per group. *D*, immunohistochemical analysis of retinas from *Rax* iCKO and control mice (P4→1 M) after light exposure using marker antibodies as follows: Rhodopsin (a rod outer segment marker), M-opsin (an M-cone outer segment marker), and S-opsin (an S-cone outer segment marker). GCL, ganglion cell layer; INL, inner nuclear layer; LED, light-emitting diode; ONL, outer nuclear layer; Rax iCKO, *Rax* tamoxifen–induced conditional KO.

To investigate the role of *Rax* in Müller glial cells in the damaged retina, *Rax* iCKO (P4→1 M) mice were exposed to light-emitting diode light (Fig. 3*A*). Light exposure, which is an accelerating factor of retinal degeneration in humans (50), induces the activation of Müller glia and associated reactive gliosis (51, 52). To evaluate histological changes, we performed immunostaining using the anti-GFAP antibody. We observed increased GFAP signals in the light-exposed control retinas compared with those in the light-unexposed control retinas, indicating that reactive Müller cell gliosis was induced by light exposure (Fig. 3, *B* and *C*). In the light-exposed *Rax* iCKO (P4→1 M) retinas, GFAP signals increased compared with those in the light-exposed control retinas (Fig. 3, *B* and *C*), suggesting that reactive gliosis induced by light exposure is enhanced by *Rax* deficiency in Müller glial cells. To examine the effects of the *Rax* deficiency in Müller glial cells on photoreceptor cells of the light-exposed retina, we immunostained retinal sections from the light-exposed *Rax* iCKO mice (P4→1 M) using marker antibodies against rhodopsin (rod outer segments), M-opsin (M-cone outer segments), and S-opsin (S-cone outer segments). No obvious differences were observed between the control and *Rax* iCKO retinas (Fig. 3*D*).

**Figure 4:**
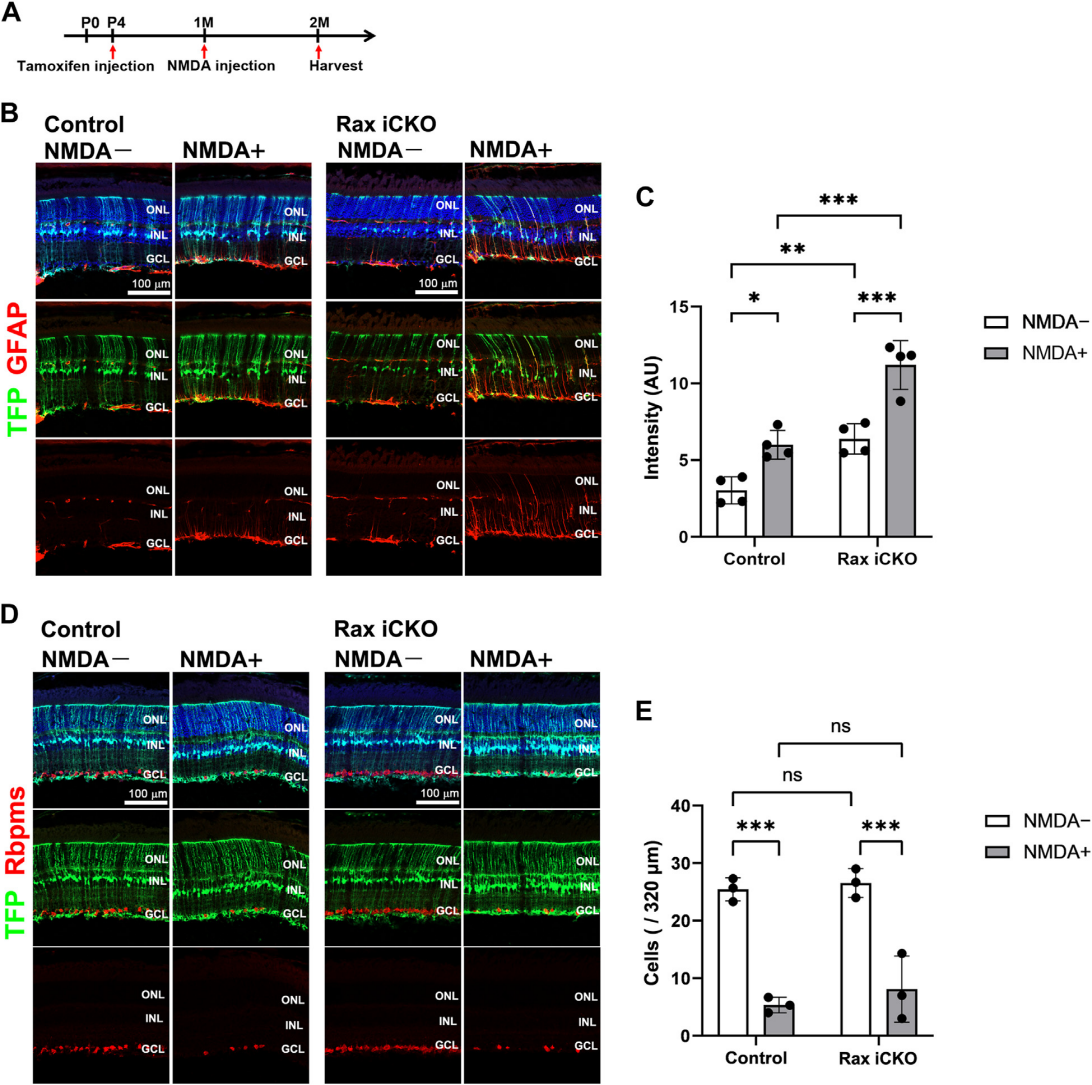
**Effects of NMDA on the *Rax* iCKO mouse retina.***A*, schedule of tamoxifen injection, NMDA injection, and mouse harvesting. Mice were injected with tamoxifen and NMDA at P4 and 1 M, respectively, and harvested at 2 M (P4→1 M→2 M). The NMDA-untreated retina was obtained from the contralateral eye without vehicle injection. *B*, immunohistochemical analysis of retinas from *Rax* iCKO and control mice (P4→1 M→2 M). Sections were immunostained using anti-GFAP (*B*) and anti-Rbpms (a ganglion cell marker) antibodies. Nuclei were stained with DAPI (*blue*). GFAP signals increased in mTFP-positive cells of the NMDA-treated *Rax* iCKO retina compared with those of the NMDA-treated control retina. *C*, the intensity of GFAP signals in the area between the ONL and GCL in the retinas of *Rax* iCKO and control mice (P4→1 M→2 M) with or without NMDA treatment. ∗*p* < 0.05, ∗∗*p* < 0.01, and ∗∗∗*p* < 0.001 (two-way ANOVA with Sidak’s multiple comparisons test). n = 4 mice per group. *D*, immunohistochemical analysis of retinas from *Rax* iCKO and control mice (P4→1 M→2 M). Sections were immunostained using anti-Rbpms. Nuclei were stained with DAPI (*blue*). *E*, quantification of the number of retinal ganglion cells detected by Rbpms immunostaining in *Rax* iCKO and control mice (P4→1 M→2 M). ∗∗∗*p* < 0.001, ns, not significant (two-way ANOVA with Sidak’s multiple comparisons test). n = 3 mice per group. DAPI, 4',6-diamidino-2-phenylindole; GCL, ganglion cell layer; INL, inner nuclear layer; NMDA, N-methyl-D-aspartic acid; ONL, outer nuclear layer; Rax iCKO, *Rax* tamoxifen–induced conditional KO.

Intravitreal injection of N-methyl-D-aspartic acid (NMDA) is a mouse experimental system that damages inner retinal neurons (53). We injected tamoxifen and NMDA into *Rax*^*flox/flox*^; *Rlbp1-CreERT2*; *R26-CAG-LoxP-mTFP1* mice at P4 and 1 M, respectively, and harvested the retina at 2 M (P4→1 M→2 M) (Fig. 4*A*). To evaluate histological changes, we performed immunostaining using the anti-GFAP antibody and observed that the GFAP signals increased in the NMDA-treated control retina compared with those in the NMDA-untreated control retina, showing that reactive Müller cell gliosis is induced by NMDA injection (Fig. 4, *B* and *C*). We also immunostained retinal sections from the NMDA-treated control eye using the anti-Rbpms (a RGC marker) antibody and observed that the number of Rbpms-positive cells significantly decreased in the NMDA-treated control retina compared with that in the NMDA-untreated control retina, indicating that RGC death was induced by NMDA treatment (Fig. 4, *D* and *E*). Although the number of Rbpms-positive cells was not significantly different between the NMDA-treated control retina and the NMDA-treated *Rax* iCKO (P4→1 M→2 M) retina, GFAP signals were stronger in the NMDA-treated *Rax* iCKO (P4→1 M→2 M) retina than in the NMDA-treated control retina, suggesting that the NMDA treatment exacerbates reactive gliosis of Müller glial cells in *Rax* iCKO mouse retinas (Fig. 4, *B*–*E*).

### Rax regulates Socs3 expression in Müller glial cells

**Figure 5:**
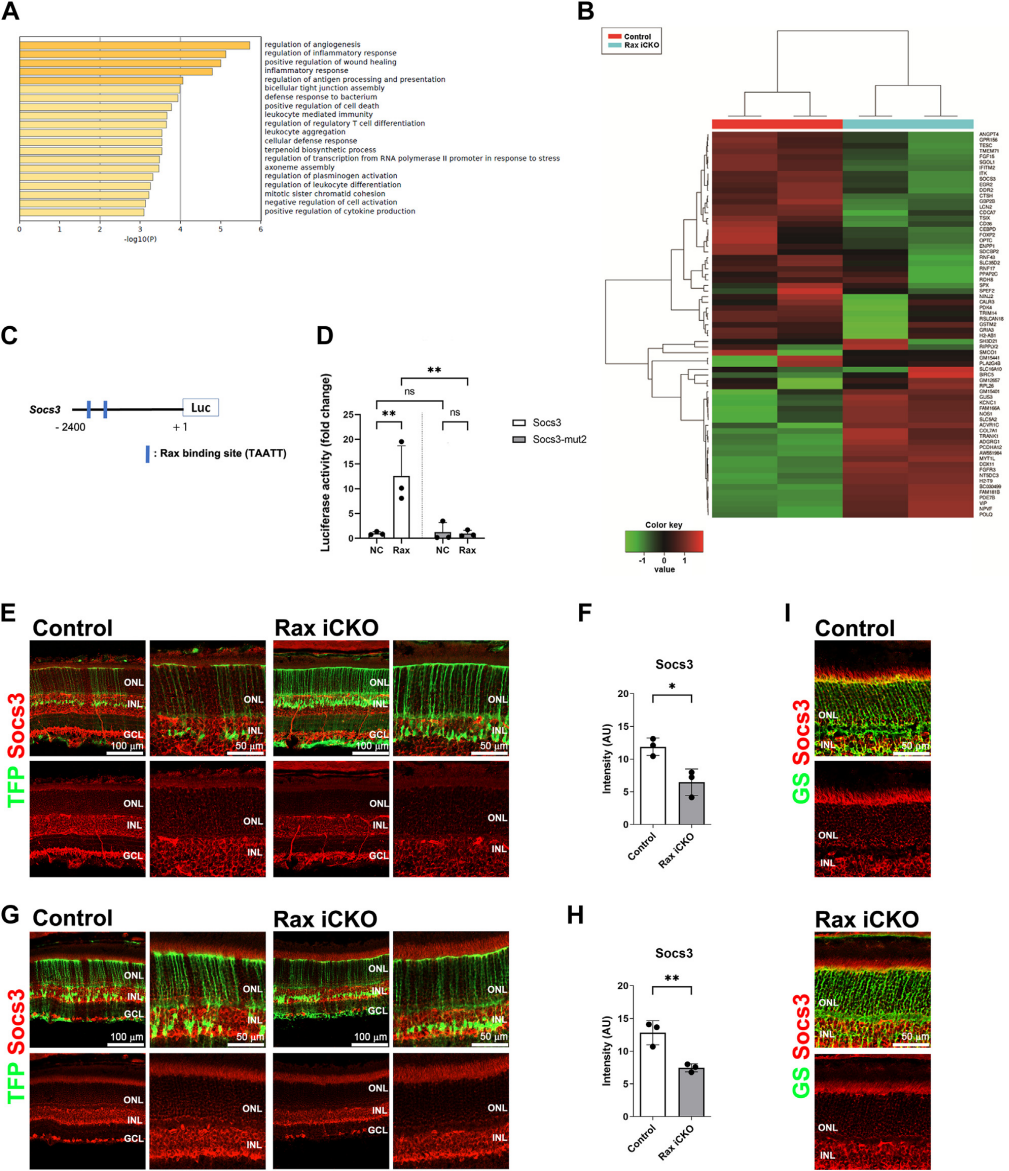
**Downregulation of *Socs3* in Müller glial cells of the *Rax* iCKO retina.***A* and *B*, RNA-seq analysis was performed in Müller glial cells of the *Rax* iCKO and control retinas (P4→P14). Müller glial cells were sorted by FACS using mTFP signals. *A*, Gene ontology (GO) enrichment analysis. The top 20 most significantly enriched biological processes determined by GO enrichment analysis for the upregulated genes (fold change >2) are shown. *B*, heatmaps of log-fold change comparisons with two biological replicates showing the 69 genes differentially expressed (average fold change >2 or < −2; *p* < 0.05, Student’s *t* test) in mTFP-positive cells from the *Rax* iCKO and control (P4→P14) retinas. The *color key* shows the log units. *C* and *D*, luciferase (Luc) reporter assay using mouse *Socs3* promoter-luciferase constructs. *C*, schematic representation of *Socs3*-promoter-luciferase constructs. The *Socs3* promoter has two predicted *Rax*-binding sites (5′-TAATT-3′). The luciferase assay promoter constructs harboring mutations in all the predicted *Rax*-binding sites (*Socs3*-mut2) were also generated. *D*, NIH 3T3 cells were cotransfected with 0.5 μg of the mouse *Socs3* promoter (positions −2400 to +1)-luciferase construct together with 0.4 μg *Rax* expression plasmids. Luciferase activity was corrected for transfection efficiency using a β-galactosidase internal control (0.1 μg) and is shown as the fold change, which was calculated as the ratio of the value for the reporter plasmid with a *Rax* expression plasmid to the value for that with an empty vector. Data are presented as mean ± SD. n = 3 experiments. ∗∗*p* < 0.01, ns, not significant (two-way ANOVA with Sidak’s multiple comparisons test). *E*, immunohistochemical analysis of retinas from *Rax* iCKO and control mice (P4→P14). Sections were immunostained with an anti-Socs3 antibody. Higher magnification images of the *left panels* are shown in the *right panels*. *F*, the intensity of Socs3 signals in the ONL of the *Rax* iCKO and control mice retinas (P4→P14). ∗*p* < 0.05 (Student’s *t* test). n = 3 mice per group. *G*, immunohistochemical analysis of retinas from 2 M *Rax* iCKO and control mice, in which tamoxifen was injected at P4. Sections were immunostained with an anti-Socs3 antibody. Higher magnification images of the *left panels* are shown in the *right panels*. *H*, the intensity of Socs3 signals in the ONL of the *Rax* iCKO and control mice retinas (P4→2 M). ∗∗*p* < 0.01 (Student’s *t* test). n = 3 mice per group. *I*, immunohistochemical analysis of retinas from 2 M *Rax* iCKO and control mice, in which tamoxifen was injected at P4. Sections were immunostained using an anti-GS (a marker of Müller glial cells) (*G*) antibody. GCL, ganglion cell layer; GS, glutamine synthetase; INL, inner nuclear layer; ONL, outer nuclear layer; Rax iCKO, *Rax* tamoxifen–induced conditional KO; Socs3, suppressor of cytokine signaling 3.

To assess the transcriptional consequences of *Rax* deficiency in Müller glial cells, we performed RNA-seq analysis using total RNA purified from mTFP-positive cells in the retinas of control and *Rax* iCKO (P4→P14) mice, which were collected by FACS. We carried out Gene Ontology (GO) enrichment analysis and classified the upregulated genes (fold change >2) into functional categories according to the GO term enrichment for biological processes. These genes were associated with several biological processes related to inflammation, including regulation of inflammatory response (Fig. 5*A*). As reactive gliosis is a general response to inflammation and is reflected by increased GFAP expression in Müller glial cells, the results of the GO enrichment analysis support the idea that Müller glial cells in the *Rax* iCKO retina undergo reactive gliosis. Using the cut-off (average fold change >2 or < −2; *p* < 0.05), we obtained 41 downregulated and 28 upregulated genes in mTFP-positive cells from the *Rax* iCKO (P4→P14) retina (Fig. 5*B*). Among the downregulated genes, we focused on *suppressor of cytokine signaling 3* (*Socs3*), which encodes a negative feedback regulator of the Janus kinase/signal transducers and activators of transcription (JAK/STAT) signaling pathway (54, 55), since a previous immunohistochemical analysis showed that *Socs3* deletion in retinal cells, including Müller glial cells, increases GFAP signals in Müller glial cells under stress (56).

To test whether Rax can transactivate *Socs3* gene, we performed a luciferase assay using the mouse *Socs3* promoter (positions −2400 to +1), which contains two predicted binding sites for Rax (Fig. 5*C*). We examined the effect of Rax expression on promoter activity in transient transfection experiments using NIH 3T3 cells. We observed that Rax transactivated the mouse *Socs3* promoter (Fig. 5*D*). To examine whether transactivation of the *Socs3* promoter by Rax depends on the predicted Rax-binding sites, we changed the predicted Rax-binding site sequence from 5′-TAATT-3′ to 5′-TGGTT-3’ (in *Socs3-mut2*) as previously described (57). When we used *Socs3-mut2* as the promoter, Rax did not transactivate the promoter (Fig. 5*D*). These results suggest that Rax directly transactivates *Socs3*.

To analyze whether Socs3 expression is regulated by Rax in Müller glial cells *in vivo*, we immunostained retinal sections from *Rax* iCKO (P4→P14 and P4→2 M) mice with antibodies against Socs3 and GS. We observed that Socs3 signals overlap with Müller glial cell processes spanning the ONL recognized by mTFP and GS signals in the control retina, suggesting that Socs3 is expressed in Mϋller glial cells (Fig. 5, *E*, *G* and *I*). We found that these Socs3 signals decreased in the *Rax* iCKO (P4→P14 and P4→2 M) retinas compared with those in the control retina (Fig. 5, *E*–*H*). These results imply that Rax may directly transactivate *Socs3* in Müller glial cells *in vivo*.

## Discussion

In the present study, we investigated the *Rax* function in Müller glial cells in the mouse retina. We deleted *Rax* in Müller glial cells and found an increased GFAP signal, a histological characteristic of reactive gliosis, in *Rax* iCKO retinas. We observed further increased GFAP signals in the *Rax* iCKO retina compared with those in the control retina following retinal injury using two different methods: light-induced damage and NMDA-induced damage. In addition, we performed RNA-seq of mTFP-positive cells isolated from *Rax* iCKO mice and investigated changes in gene expression. We found that the expression of *Socs3* was significantly reduced in Müller glial cells of the *Rax* iCKO retina compared with that of the control retina. Our reporter gene assay showed that Rax directly transactivates *Socs3*, suggesting that the Rax–Socs3 axis plays an important role in maintaining homeostasis of Müller glial cells in the postnatal mouse retina.

We have previously reported that the homeobox-containing gene *Rax* is expressed at the embryonic stage and is responsible for optic vesicle formation, retinal cell proliferation, retinal cell fate determination, and photoreceptor maturation and maintenance (22, 24, 25, 35, 36). To further investigate the *Rax* function in retinal development, we performed retroviral lineage analysis using a murine replication-incompetent retrovirus LIA that only infects progenitor cells (Fig. 1). LIA-Cre-infected *Rax*^*flox/flox*^ mouse retinas, which were deficient for *Rax* in progenitor cells, showed a significant increase in the percentage of rod photoreceptors and amacrine cells and a significant reduction in the percentage of bipolar and Müller glial cells (Fig. 1*B*). In contrast, LIA-Rax-infected WT mouse retinas, in which *Rax* was overexpressed in progenitor cells, exhibited a significant decrease in the percentage of rod photoreceptors and a significant increase in the percentage of bipolar and Müller glial cells (Fig. 1*F*). Most rod photoreceptors and some amacrine cells differentiate early in the postnatal period, while bipolar and Müller glial cells differentiate late in the postnatal period (58, 59). The increase in the percentage of rod photoreceptors and amacrine cells and the decrease in the percentage of late-born retinal cell types, bipolar, and Müller glial cells in the LIA-Cre-infected (*Rax*-deficient) retina are considered to be due to the loss of pluripotency and precocious differentiation of progenitor cells. Notably, the percentage of bipolar and Müller glial cells significantly increased, whereas the percentage of rod photoreceptors significantly decreased in LIA-Rax-infected (Rax-overexpressing) retinas. These results suggest that Rax is required for maintaining proper cell differentiation competency of progenitor cells, which generate late-born retinal cell types, including Müller glial cells, in the postnatal mouse retina. We speculate that as *Rax* expression gradually declines in progenitor cells after birth, progenitor cells that retain relatively high levels of Rax are inclined to differentiate into Müller glial cells. This may be consistent with the report that Müller glial cells exhibit a gene expression profile similar to that of progenitor cells (37, 38, 39). Although human *RAX* mutations are associated with anophthalmia, microphthalmia, coloboma, and sclerocornea (29, 60), the mechanisms by which these mutations cause human diseases are poorly understood. Future retroviral lineage analysis by overexpression of the human *RAX* gene harboring these mutations would contribute to the understanding of the underlying pathological mechanisms. The results of our lineage analysis were consistent with those of our previous report, showing that Rax transduction leads to gliogenesis. In contrast, a previous report demonstrated that Rax overexpression using electroporation induces photoreceptor generation and suppresses gliogenesis (61). This phenotypic difference may reflect the amount of transduced *Rax* due to differences in the methods used.

We previously reported that the Rax localization gradually shifts to Müller glial cells from photoreceptor cells after postnatal photoreceptor differentiation and maturation in the mouse retina (36). To investigate the *in vivo* function of *Rax* in Müller glial cells from postnatal development to mature stages, we treated *Rax*^*flox/flox*^; *Rlbp1-CreERT2*; *R26-CAG-LoxP-mTFP1* mice with tamoxifen at P4 and harvested the retinas at P14 and 1 M (P4→P14 and P4→1 M) (Fig. 2, *B* and *H*). We observed increased GFAP signals, a histological characteristic of reactive Müller cell gliosis, in mTFP-positive cells of *Rax* iCKO (P4→P14 and P4→1 M) retinas (Fig. 2, *E*, *F*, *I* and *J*). Next, to investigate whether the increase in GFAP signals in Müller glial cells of the *Rax* iCKO retina is influenced by the timing of tamoxifen administration, we injected tamoxifen into the mice at 1 M and harvested the retina from them at 2 M (1 M→2 M) (Fig. S4*A*). In contrast to the observations in the *Rax* iCKO (P4→P14 and P4→1 M) retina, we did not observe a substantial increase in GFAP signals in the mTFP-positive cells of the *Rax* iCKO (1 M→2 M) retina (Fig. S4*B*). We also injected tamoxifen into the mice at P9 and P12 and harvested the retinas at 1 M (P9 or P12→1 M) (Fig. S4*M*). We did not observe an increase in GFAP signals in the mTFP-positive cells of the *Rax* iCKO (P9 or P12→1 M) retinas (Fig. S4*N*). Given that retinogenesis is completed around P7 (47, 48, 49), these results suggest that the *Rax* deficiency in differentiating Müller glial cells induces reactive gliosis in the retina.

Considering the observation of increased GFAP signal, a histological characteristic of reactive gliosis, in the *Rax* iCKO retina, whose ability to control inflammation is predicted to be decreased, we next examined the effects of light-induced damage and NMDA-induced damage to *Rax* iCKO retinas (Figs. 3 and 4). We observed enhanced GFAP signals in the *Rax* iCKO retina compared with those in the control retina of both retinal damage models (Figs. 3, *B* and *C*, and 4, *B* and *C*). These results suggest that *Rax* in Müller glial cells confers resistance to retinal inflammation. In the present study, increased GFAP signals in the retina, induced by postnatal *Rax* deficiency, were observed up to 2 M, with no accompanying functional or structural changes under normal and stress conditions (Figs. S2, *B–H*, S3, 3 and 4). However, we cannot rule out the possibility that functional or structural changes may occur in later periods because of the prolonged inflammatory state caused by *Rax* deficiency in Müller glial cells. In addition, it is possible that we could not detect these changes in the *Rax* iCKO retina due to limitations of the methods and/or *Rax* deletion in not all Müller glial cells. Future studies utilizing other histological and electrophysiological analyses in combination with behavioral analyses may reveal retinal changes resulting from *Rax* deficiency in Müller glial cells.

In FACS-purified mTFP-positive cells deficient in *Rax*, we detected changes in the expression of various genes by RNA-seq analysis (Fig. 5, *A* and *B*). GO enrichment analysis indicated that the genes upregulated by *Rax* depletion were associated with several biological processes related to inflammation (Fig. 5*A*). Among the downregulated genes, we focused on *Socs3*, since a previous immunohistochemical analysis showed that the *Socs3* deletion in retinal cells, including Müller glial cells, increased GFAP signals in Müller glial cells under stress (Fig. 5*B*) (56). The Socs3 protein can bind to and inhibit the activity of JAKs and can selectively inhibit interleukin-6 (IL-6) signaling by binding to the IL-6 receptor gp130 (62, 63, 64). IL-6 signaling *via* the gp130 receptor causes tissue dysfunction in various organs including the retina (65, 66). Socs3 is expressed in the adult retina, and its expression is most prominent in photoreceptor cells and Müller glial cells under the condition of LPS-induced inflammation (67). The predicted Rax-binding sequence, 5′-TAATT-3′, is present at two locations on −2.4 kb upstream of the transcription initiation site of *Socs3* (Fig. 5*C*) and, in fact, we observed that Rax transactivates the mouse *Socs3* promoter (Fig. 5*D*). We immunostained the *Rax* iCKO (P4→P14 and P4→2 M) retinas and found that Socs3 signals overlapped with Müller glial cell processes in the ONL decrease in the *Rax* iCKO (P4→P14 and P4→2 M) retinas compared with those in the control retina (Fig. 5, *E*–*I*). Notably, it was previously reported that the activated JAK/STAT pathway can upregulate GFAP expression *via* direct binding of phosphorylated STAT3 dimers to the *Gfap* promoter in Müller glial cells (68). Conclusively, our results imply that Rax may suppress GFAP expression by directly transactivating *Socs3* in Müller glial cells *in vivo*. There are previous reports showing that mice lacking *Socs3* in the retina showed delayed photoreceptor differentiation (69, 70). We have previously reported that Rax plays an essential role in photoreceptor maturation and maintenance (36). Considering the current observations in Müller glial cells, the mechanism of the Rax-Socs3 axis may be conserved in developing and mature photoreceptors. However, we cannot exclude the possibility that other factor(s) are involved in the increased expression of GFAP in Müller glial cells in the *Rax* iCKO retina. In our RNA sequencing analysis, we noticed that *CCAAT/enhancer-binding* *protein delta* (*Cebpd*) is also downregulated in Müller glial cells from the *Rax* iCKO retina (Fig. 5*B*). *Cebpd* is the gene that encodes one of the CEBPs family of proteins, whose functions are known as transcription factors in cellular differentiation (71, 72, 73), metabolism (74) and immune responses (75). Among the CEBPs family members, CCAAT/Cebp beta has been reported to transactivate *Socs3* (76). Given that the predicted DNA-binding sequences in the CEBPs family are almost the same (77, 78) and that Cebpd and Cebp beta can bind to the same DNA site (75, 79), these previous reports suggest that Rax transactivates *Socs3* but also *Cebpd* to suppress reactive Müller cell gliosis. Further studies are needed to elucidate the molecular mechanisms underlying Rax-mediated suppression of GFAP expression in Müller glial cells.

In *Rax* iCKO (P4→P14, P4→1 M, and P4→2 M) retinas, GFAP signals, a marker of reactive gliosis, were found to be upregulated (Figs. 2, *E*, *F*, *I*, and *J*, 3, *B* and *C* and 4, *B* and *C*). Reactive gliosis is induced by various types of physical and chemical damages. Severe and prolonged reactive gliosis is usually associated with reduced neuronal viability (80). Reactive gliosis is associated with several retinal diseases, including photic damage, retinal trauma, ischemia, retinal detachment, glaucoma, diabetic retinopathy, and age-related macular degeneration (81, 82). All of these retinal diseases have been reported to display increased GFAP protein levels in Müller glial cells (83, 84, 85, 86, 87, 88, 89). The increased expression of GFAP and downregulation of Socs3 observed in *Rax* iCKO mice together with the luciferase assay results suggest that the Rax homeoprotein is involved in the pathogenesis and progression of these retinal diseases to suppress inflammation in Müller glial cells by directly transactivating *Socs3*. It was previously reported that Lhx2 in Müller glial cells protects the retina from retinal inflammation (90).Whether and how Lhx2 and Socs3 are related remains to be clarified. Previous ChIP analysis of the developing mouse retina showed that Lhx2 binds to the *Rax* promoter at P2 and P8, suggesting that Rax is downstream of Lhx2 in Müller glial cells (46). Here, we propose a functional role for the *Rax*-*Socs3* axis in suppressing retinal inflammation. Further studies are needed to advance our understanding of the protective mechanisms against retinal inflammation in Müller glial cells.

## Experimental procedures

### Animal care

All the procedures conformed to the ARVO statement for the Use of Animals in Ophthalmic and Vision Research. These procedures were approved by the Institutional Safety Committee on Recombinant DNA Experiments (approval ID 04913) and Animal Experimental Committees of the Institute for Protein Research (approval ID R04-02-0) at Osaka University and were performed in compliance with the institutional guidelines. Mice were housed in a temperature-controlled room at 22 °C with a 12 h light/dark cycle. Freshwater and rodent diets were always available. All animal experiments were performed with mice of either sex.

### Plasmid constructs

We subcloned a 2.4-kb upstream genomic fragment of the mouse *Socs3* gene (bp −2400 to +1) into the pGL3-Basic vector (Promega) to generate the pGL3b-mouse Socs3-luc reporter plasmid. Mutations in the predicted *Rax*-binding sites (5′-TAATT-3′) (35, 57) of the mouse Socs3 promoter were introduced by PCR with mutated PCR primers. The resulting construct was named pGL3b-mouse Socs3-mut2-luc. The construction of the *Rax* expression vector (pME18S-Rax) has been previously described (36). We constructed LIA-Rax and LIA-Cre vectors by inserting a mouse *Rax* complementary DNA (cDNA) fragment or *Cre* fragment into the pLIA vector.

### Cell composition analysis using retrovirus

*In vivo* infection was carried out by injection of the retrovirus into P0 mouse retinas. The infected retinas were harvested at P28, fixed, and stained for AP. The procedures for sectioning and counting infected clones were performed as described previously (91). Cell types are determined by the characteristic morphology and location of terminally differentiated cells (1, 42). More than 600 clones from at least three retinas were counted for each virus.

### Generation of *Rax*^*flox/flox*^; *Rlbp1-CreERT2* mice

*β-gal* in the *pβ-gal-Basic* vector (Clontech) was replaced with the *CreERT2* fragment, which was excised from *pCre-ERT2* (92). The ∼3.1 kb promoter fragment of the *Rlbp1* gene (44) was inserted upstream of *CreERT2* and the insulator-insulator fragment, which was excised from *pJC13-1* (93), was inserted downstream of *CreERT2* to generate a *pRlbp1-CreERT2-Basic* vector. The *Rlbp1-CreERT2-insulator-insulator* fragment was injected into the pronuclei of fertilized single-cell eggs of B6C3F1 mice. *Rlbp1-CreERT2* mice were backcrossed with 129 Sv/Ev mice to generate homozygous variants encoding Leu450 in *Rpe65* (94). To observe Cre recombinase activity induced by tamoxifen injection in *Rlbp1-CreERT2* transgenic mice, we crossed them with *R26-CAG-LoxP-mTFP1* reporter strain (#RBRC05147, RIKEN BRC) (45) which was also backcrossed with 129 Sv/Ev mice. *Rlbp1-CreERT2*; *R26-CAG-LoxP-mTFP1* mice were treated with tamoxifen to analyze mTFP1 expression. *Rax*^*flox/flox*^ mice (129 Sv/Ev background) were generated as described in our previous study (35). *Rax*^*flox/flox*^ mice were mated with *Rlbp1-CreERT2*; *R26-CAG-LoxP-mTFP1* mice.

### Tamoxifen treatment

Tamoxifen (Sigma) was dissolved in sunflower seed oil (Sigma) to a concentration of 10 mg/ml and injected 0.2 mg or 0.4 mg of it intraperitoneally into mice at postnatal day 4 (P4), P9, P12, or 1 month of age (1 M).

### Cell dissociation and FACS

Whole retinas were dissociated into cell suspensions by incubation with papain (9 U/sample) (Nacalai Tesque) for 15 min at 37 °C. The reaction was stopped by the addition of Dulbecco's Modified Eagle's Medium (DMEM) (Sigma) containing 10% fetal bovine serum. Suspended cells were incubated with DNase I at 37 °C for 5 min. Cells were pelleted by centrifugation, resuspended in 1 ml DMEM, and immediately FACS-sorted. TFP-positive and TFP-negative cells were sorted using a BD FACSAriaIIu flow cytometer (BD Biosciences) with a 488 nm laser and FITC fluorescence filter. Cells were collected for postsorting FACS analysis. For RNA-seq or RT-PCR, TFP-positive cells were collected separately from the FACS sheath fluid and RNA was immediately extracted.

### RT-PCR analyses

RT-PCR analyses were performed as previously described (95). Total RNAs were extracted using TRIzol LS (Ambion) from Müller glial cells purified by FACS from control and *Rax* iCKO retinas treated with tamoxifen (P4→P14). Total RNA (30 ng) was reverse-transcribed into cDNA with random hexamers using PrimeScript II reagent (TaKaRa). The cDNAs were used for PCR with rTaq polymerase (TaKaRa). Primer sequences used for amplification were as follows: Rax, F, 5′-ACTCGAAGCTGT CGGAGGAGGAACCTC-3′ and R, 5′-ACTTCCAGTTTCTCCTGGCGCCTCCAC-3′; β-actin, F, 5′-CGTGCGTGACATCAAAGAGAA-3′, and R, 5′-TGGATGCCACAGGAT TCCAT-3′.

### Immunofluorescent analysis of retinal sections

Immunohistochemical analysis of retinal sections was performed as previously described (96). Mouse eyes or eye cups were fixed with 4% paraformaldehyde in PBS for 5 or 30 min at room temperature. The samples were rinsed in PBS, followed by cryoprotection using 30% sucrose in PBS overnight at 4 °C, embedded in TissueTek optimal cutting temperature compound 4583 (Sakura), frozen, and sectioned. Frozen 20 μm sections on slides were dried for more than 3 h at room temperature, rehydrated in PBS for 5 min, incubated with blocking buffer (5% normal donkey serum and 0.1% Triton X-100 in PBS) for 1 h, and then incubated with primary antibodies for 4 h at room temperature or overnight at 4 °C. Slides were washed with PBS three times for 5 min each and incubated with fluorescent dye-conjugated secondary antibodies and 4',6-diamidino-2-phenylindole (1:1000, Nacalai Tesque) for 2 h at room temperature while shielded from light. After washing three times with PBS, the sections were coverslipped with Gelvatol. The specimens were observed under a laser confocal microscope (LSM700 or 900, Carl Zeiss). The primary antibodies used in this study were guinea pig anti-Rax (1:500) (36), goat anti-Lhx2 (1:1000, Santa Cruz, sc-19342), rabbit anti-Rhodopsin (1:2,000, LSL, LB-5597), goat anti-S-opsin (1:100, Santa Cruz, sc-14363), rabbit anti-M-opsin (1:500, Millipore, AB5405), rabbit anti-Chx10 (1:500) (97), mouse anti-Pax6 (1:250, DSHB), rabbit anti-Sox9 (1:500, Millipore, AB5535), rabbit anti-Calbindin (1:1000, Calbiochem, PC253L), mouse anti-S100β (1:2,500, Sigma, S-2532), guinea pig anti-Rbpms (1:1000, Millipore, ABN1376), mouse anti-GFAP (1:500, Sigma, G3893), mouse anti-Socs3 (1:100, Abcam, SO1) (98, 99), rabbit anti-Vimentin (1:500, Abcam EPR3776), and rabbit anti-GS (1:500, Sigma, G2781). Cy3-conjugated (1:500, Jackson ImmunoResearch Laboratories), Alexa Fluor 488–conjugated (1:500, Sigma), and DyLight 649–conjugated (1:500, Jackson) secondary antibodies were used. Immunofluorescence signal intensities were measured and quantified using the National Institutes of Health (NIH) ImageJ software (https://imagej.nih.gov/ij/index.html) under the blinded condition for mouse genotypes.

### ERG recordings

ERGs were recorded as previously described (100). Briefly, mice were dark-adapted overnight and then anesthetized with an intraperitoneal injection of 100 mg/kg ketamine and 10 mg/kg xylazine diluted in saline (Otsuka). The pupils were dilated using topical 0.5% tropicamide and 0.5% phenylephrine HCl. The ERG responses were measured using a PuREC system with light-emitting diode (LED) electrodes (Mayo Corporation). Mice were placed on a heating pad and stimulated with an LED flash. Four levels of stimulus intensities ranging from −4.0 to 1.0 log cd s m^−2^ were used for the scotopic ERGs. After the mice were light-adapted for 10 min, the photopic ERGs were recorded on a rod-suppressing white background of 1.3 log cd s m^-2^. Four levels of stimulus intensities ranging from −0.5 to 1.0 log cd s m^−2^ were used for the photopic ERGs. Eight responses at −4.0 log cd s m^−2^ and four responses at −3.0 log cd s m^−2^ were averaged for the scotopic recordings. Sixteen responses were averaged for the photopic recordings.

### Exposure to LED light

The exposure of mice to LED light was repeated for 6 days, as described previously (101). After dark adaptation for 24 h (day 1) or 12 h (from day 2), mouse pupils were dilated with 1% cyclopentolate hydrochloride eye drops (Santen Pharmaceuticals) 30 min before exposure to LED light (∼450 nm). Nonanesthetized mice were exposed to ∼9000 l× LED light for 3 h in a mirrored device separated individually by clear partitions for light reflection. The ambient temperature during light exposure was maintained at 25 °C ± 1.5 deg. C. After exposure to LED light, the mice were returned to their cages and housed under normal light conditions for 9 h before the next dark adaptation. After the last exposure to LED light, the mice were returned to their cages and housed in a normal 12 h dark/light cycle. Retinal dissection was performed on day 7. *Rax*^*flox/flox*^ or *Rax*^*flox/+*^; *Rlbp1-CreERT2*; *R26-CAG-LoxP-mTFP1* mice were backcrossed with 129 Sv/Ev mice to generate *Rax*^*flox/flox*^ and *Rax*^*flox/+*^; *Rlbp1-CreERT2*; *R26-CAG-LoxP-mTFP1* mice homozygous for the variant encoding Leu450 in *Rpe65*, which were used for the experiment (94).

### NMDA treatment

NMDA (Nacalai Tesque) was dissolved in saline to a concentration of 10 mM and 2 μl of NMDA solution was injected intravitreally into mice at 1 month of age (1 M).

### RNA-seq and data analysis

RNA-seq analysis was performed as previously described (102) with some modifications. Müller glial RNAs from control and *Rax* iCKO mice (P4→P14) were sorted by FACS using mTFP signals and isolated using TRIzol LS RNA extraction reagent (Invitrogen). Sequencing was performed on an Illumina NovaSeq 6000 platform in 101-base single-end mode. Raw reads were mapped to mouse reference genome sequences (mm10) using TopHat ver. 2.0.13, in combination with Bowtie2 ver. 2.3.5.1 and SAMtools ver. 1.11. The number of fragments per kilobase of exon per million mapped fragments was calculated using Cufflinks ver. 2.2.1. Heatmap visualization was conducted using the iDEP web tools (103) with default parameters. GO analysis was performed using the web tool Metascape (104). The *Rax* gene was excluded from the GO analyses.

### Cell culture and transfection

NIH 3T3 cells (JCRB Cell Bank) were cultured in DMEM (Sigma) containing 10% calf serum supplemented with penicillin (100 μg/ml) and streptomycin (100 μg/ml) at 37 °C with 5% CO_2_. Transfection was performed with Lipofectamine 3000 (Invitrogen).

### Luciferase assay

The luciferase assay was performed as previously described (105). We transfected 0.5 μg of luciferase reporter plasmid DNAs (pGL3b-mouse Socs3-luc or pGL3b-mouse Socs3-mut2-luc) and 0.4 μg of mouse *Rax* expression vector DNAs (pME18S or pME18S-Rax) per well into NIH 3T3 cells in a 12-well plate using Lipofectamine 3000 (Invitrogen). A β-galactosidase expression vector (β-SV; Promega) was co-transfected to normalize the transfection efficiency. After transfection, cells were incubated for 48 h and lysed with reporter lysis buffer (Promega). Luciferase activity was measured using a firefly luciferase assay system (Promega), according to the manufacturer’s protocol. The luminescence signal was detected using the GloMax-Multi+ detection system (Promega).

### Statistical analysis

Data are represented as the mean ± SD. Statistical analysis was performed using the Student’s *t* test, two-way ANOVA with Sidak’s multiple comparisons test, or two-way repeated measures ANOVA, as indicated in the Figure legends. Differences were considered statistically significant at *p* < 0.05.

## Data availability

All sequencing data are available on GEO (GSE237650).

## Supporting information

This article contains supporting information.

## Conflict of interest

The authors declare that they have no conflicts of interest with the contents of this article.
