# Supplementary material for: The Rax homeoprotein in Müller glial cells is required for homeostasis maintenance of the postnatal mouse retina
Source: J Biol Chem. 2023 Nov 15;299(12):105461. doi: 10.1016/j.jbc.2023.105461 (PMC10714373; doi:10.1016/j.jbc.2023.105461)
Supplement: Supplemental Fig. S1–S4 [file mmc1.docx]

**Supporting Information**

The Rax homeoprotein in Müller glial cells is required for homeostasis maintenance of the postnatal mouse retina

Takuya Yoshimoto^1,2,#^, Taro Chaya^1,#^, Leah R. Varner^1^, Makoto Ando^1^, Toshinori Tujii^1^, Daisuke Motooka^3^, Kazuhiro Kimura^2^, Takahisa Furukawa^1,*^

^#^These authors contributed equally to this work.

^1^Laboratory for Molecular and Developmental Biology, Institute for Protein Research, Osaka University, 3-2 Yamadaoka, Suita, Osaka, 565-0871, Japan.

^2^Department of Ophthalmology, Yamaguchi University Graduate School of Medicine, Yamaguchi University, 1-1-1 Minami-kogushi, Ube, Yamaguchi, 755-8505, Japan.

^3^Genome Information Research Center, Research Institute for Microbial Diseases, Osaka University, Osaka 565-0871, Japan

**Corresponding author:**

^*^Author for correspondence

E-mail: takahisa.furukawa@protein.osaka-u.ac.jp

Laboratory for Molecular and Developmental Biology, Institute for Protein Research, Osaka University, 3-2 Yamadaoka, Suita, Osaka, 565-0871, Japan

Phone: +81-6-6879-8631

Fax: +81-6-6879-8633

**Material included:**

Fig. S1: Generation of *Rlbp1-CreERT2* transgenic mice.

Fig. S2: RT-PCR and histological analysis of the *Rax* iCKO retina.

Fig. S3: ERG analysis of *Rax* iCKO mice.

Fig. S4: Phenotypic analysis of *Rax* iCKO mice in which tamoxifen was injected later than P4.

**Figure S1. Generation of *Rlbp1-CreERT2* transgenic mice.**


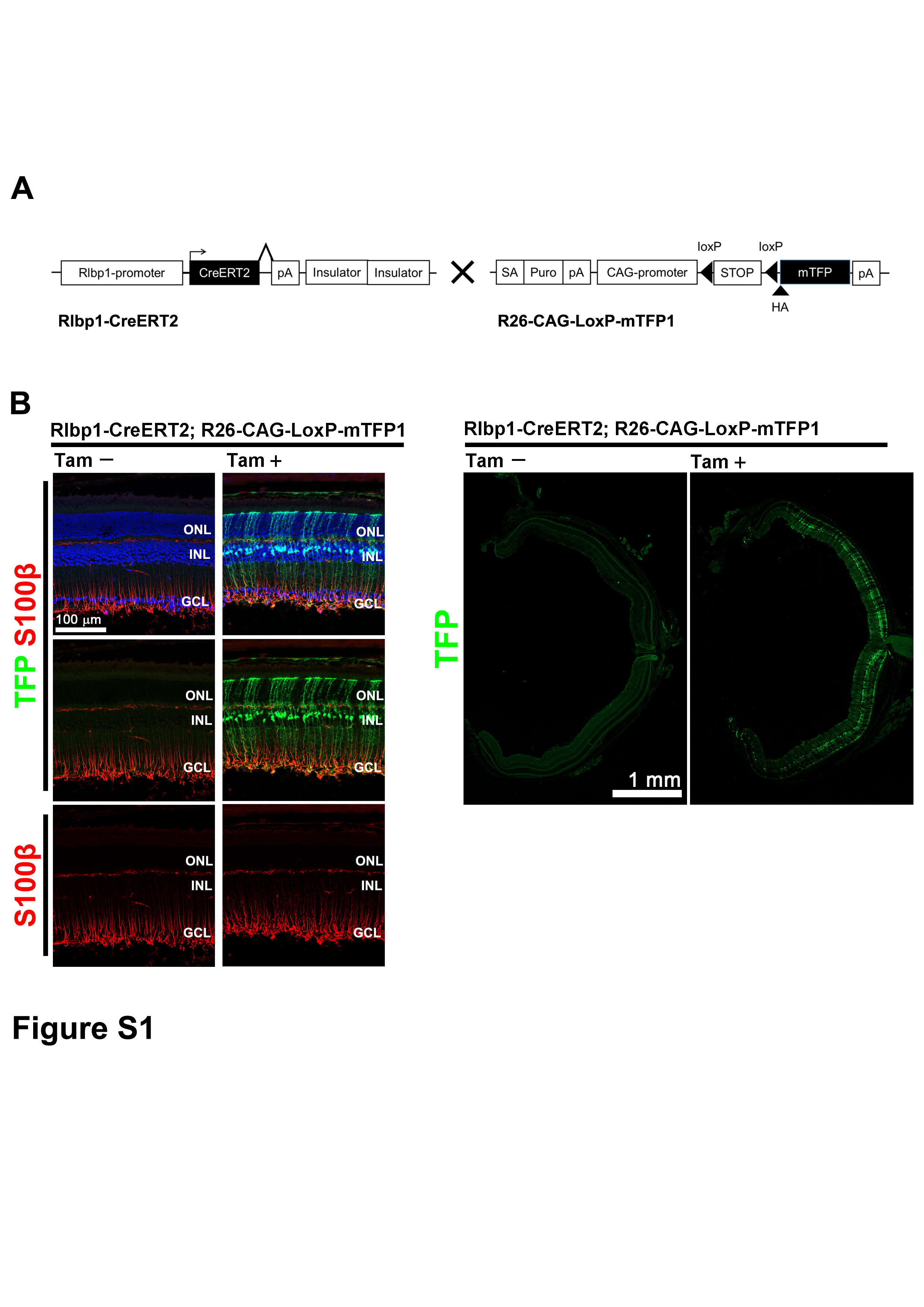


**(A)** Structure of Rlbp1 3.1kb-promoter-driven CreERT2 (Rlbp1-CreERT2) construct, and R26-CAG-LoxP-mTFP1 construct, in which the expression of Cre-mediated mTFP1 is induced under the control of the CAG promoter.

**(B)** Immunohistochemical analysis of retinas in Rlbp1-CreERT2; R26-CAG-LoxP- mTFP1 mice with or without tamoxifen injection. Sections were immunostained with an anti-S100β antibody (a Müller glial cell marker). Nuclei were stained with DAPI (blue).

ONL, outer nuclear layer; INL, inner nuclear layer; GCL, ganglion cell layer.


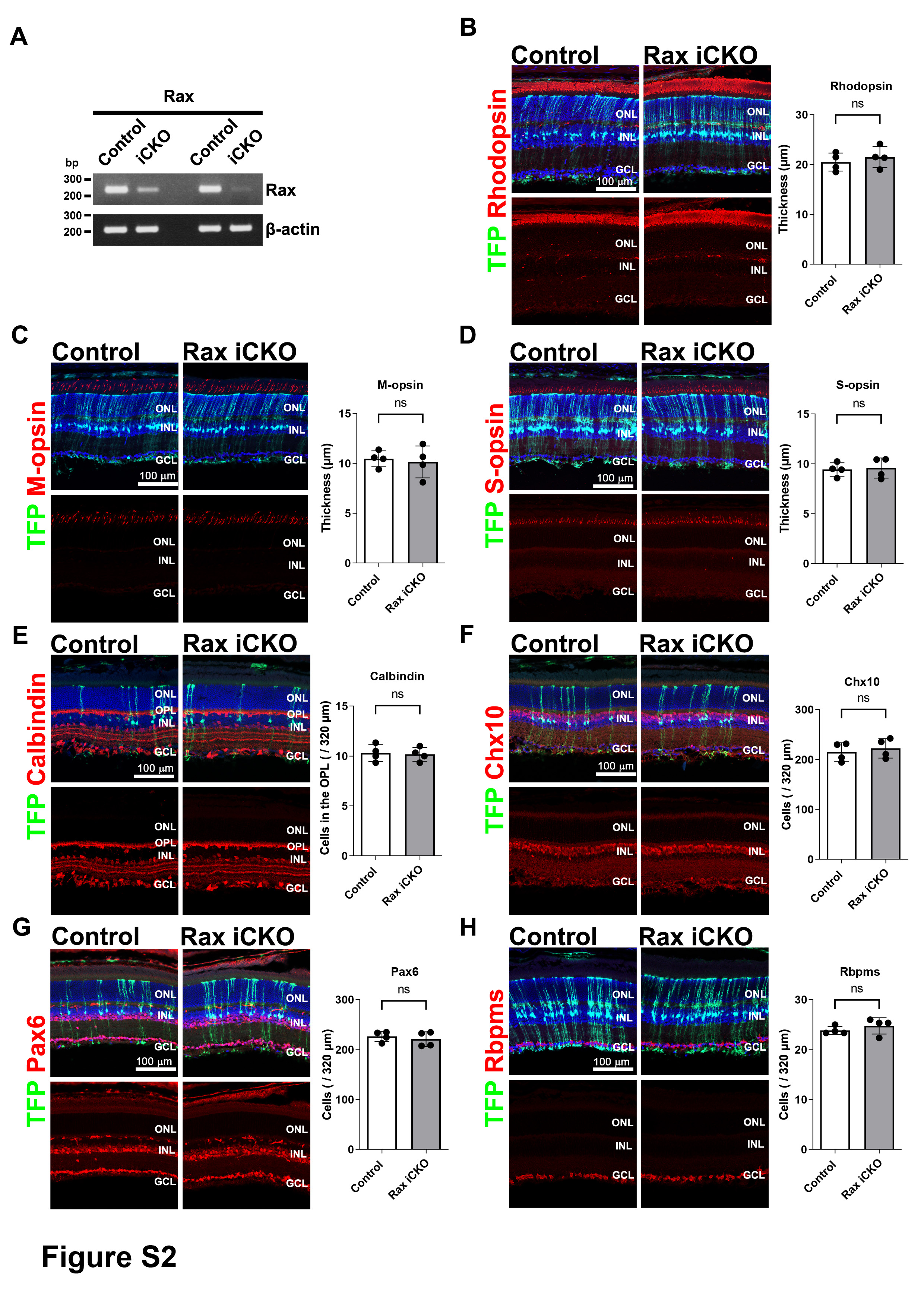


**Figure S2. RT-PCR and histological analysis of the *Rax* iCKO retina.**

**(A)** RT-PCR analysis of *Rax* transcripts in mTFP-positive cells sorted by FACS from the retinas of two additional pairs of control and *Rax* iCKO mice (P4→P14). *β-actin* was used as a loading control.

**(B-H)** Retinal sections from control and Rax iCKO mice (P4→1M) were immunostained with anti-Rhodopsin (a rod outer segment marker) (B), anti-M-opsin (an M-cone outer segment marker) (C), anti-S-opsin (an S-cone outer segment marker) (D), anti-Calbindin (a marker for horizontal cells and a part of amacrine cells) (E), anti-Chx10 (a bipolar cell marker) (F), anti-Pax6 (an amacrine and ganglion cell marker) (G), and anti-Rbpms (a ganglion cell marker) (H) antibodies. Nuclei were stained with DAPI (blue). The thickness of photoreceptor outer segments stained with antibodies against Rhodopsin, M-opsin, and S-opsin were measured (B-D). The number of marker-positive cells was counted (E-H). ns, not significant (Student’s *t*-test). n = 4 mice per group. ONL, outer nuclear layer; OPL, outer plexiform layer; INL, inner nuclear layer; GCL, ganglion cell layer.

**Figure S3. ERG analysis of *Rax* iCKO mice.**


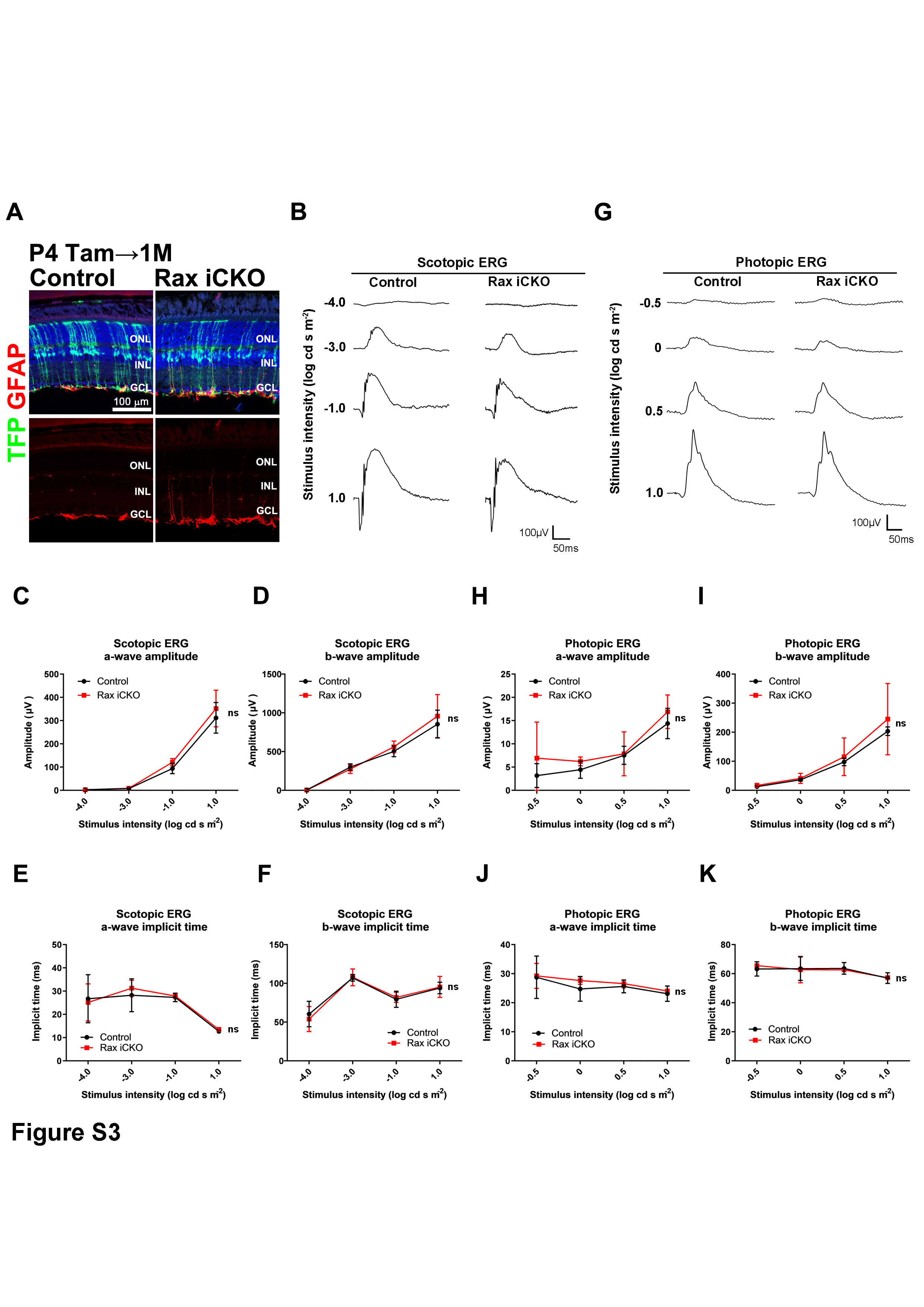


**(A)** Retinal sections from the control and *Rax* iCKO mice (P4→1M) used for ERG analysis were immunostained using an anti-GFAP antibody. Nuclei were stained with DAPI (blue). ONL, outer nuclear layer; INL, inner nuclear layer; GCL, ganglion cell layer.

**(B-F)** Scotopic ERGs were recorded from *Rax* iCKO and control mice (P4→1M) (n = 3 and 4 mice, control and *Rax* iCKO, respectively). (B) Representative scotopic ERGs elicited by four different stimulus intensities (-4.0 to 1.0 log cd s/m^2^) from *Rax* iCKO and control mice are presented. (C, D) The scotopic amplitudes of a- (C) and b-waves (D) are shown as a function of the stimulus intensity. (E, F) The scotopic implicit time of a- (E) and b-waves (F) are shown as a function of the stimulus intensity. Data are presented as mean ± SD. n = 3 and 4 mice, control and *Rax* iCKO, respectively. ns, not significant (two-way repeated-measures ANOVA).

**(G-K)** Photopic ERGs were recorded from *Rax* iCKO and control mice (P4→1M) (n = 3 and 4 mice, control and *Rax* iCKO, respectively). (G) Representative photopic ERGs elicited by four different stimulus intensities (-0.5 to 1.0 log cd s/m^2^) from *Rax* iCKO and control mice are presented. (H, I) The photopic amplitudes of a- (H) and b-waves (I) are shown as a function of the stimulus intensity. (J, K) The scotopic implicit time of a- (J) and b-waves (K) are shown as a function of the stimulus intensity. Data are presented as mean ± SD. n = 3 and 4 mice, control and *Rax* iCKO, respectively. ns, not significant (two-way repeated-measures ANOVA).


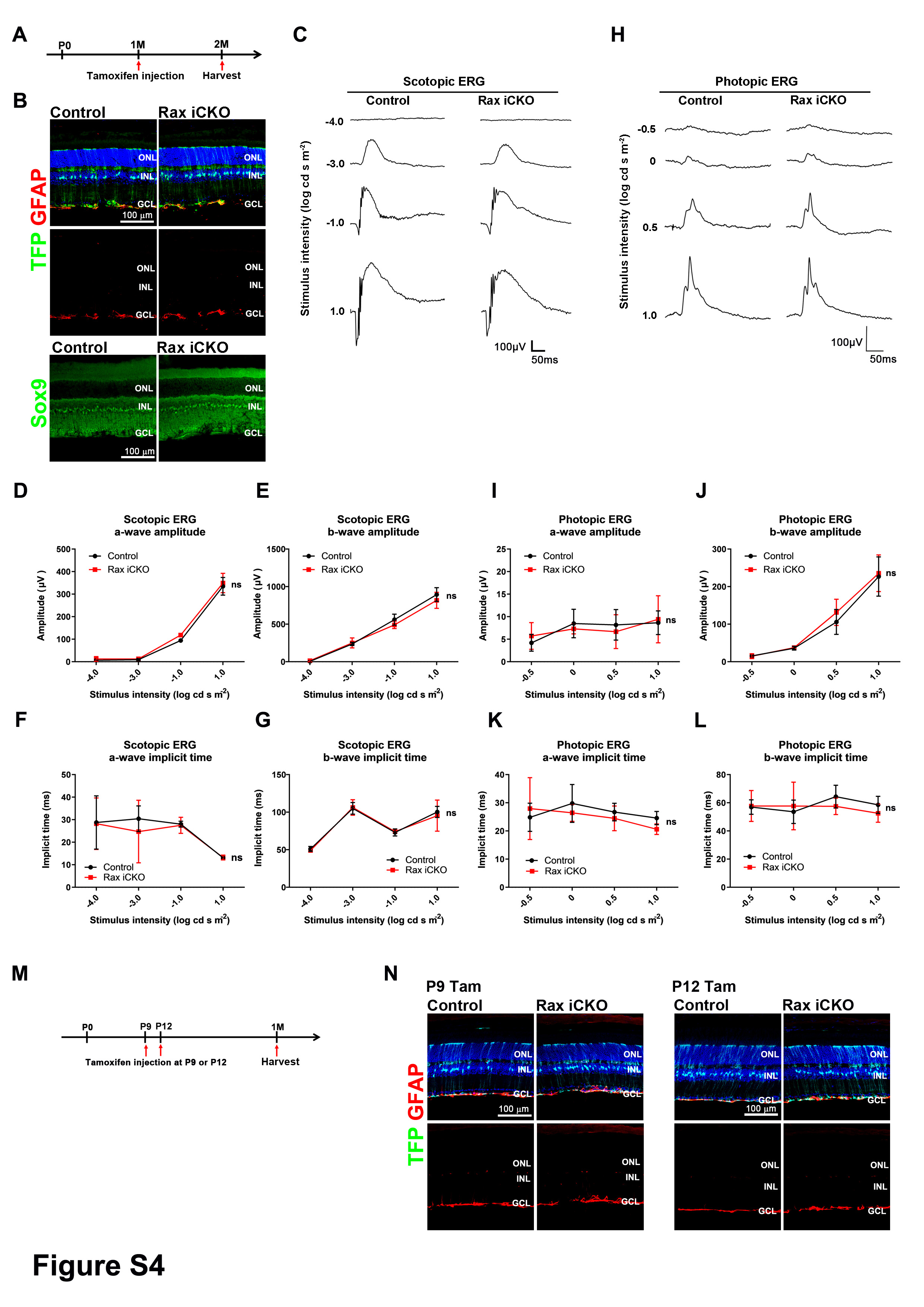


**Figure S4. Phenotypic analysis of *Rax* iCKO mice in which tamoxifen was injected later than P4.**

**(A)** Schedule for tamoxifen injection and harvest of mice. Mice were injected with tamoxifen at 1M and harvested at 2M (1M→2M).

**(B)** Immunohistochemical analysis of retinas from *Rax* iCKO and control mice (1M→2M) used for ERG analysis. Retinal sections were immunostained with anti-GFAP and anti-Sox9 antibodies. Nuclei were stained with DAPI (blue).

**(C-L)** ERGs were recorded from *Rax* iCKO and control mice (1M→2M) (n = 3 per each genotype). (C) Representative scotopic ERGs elicited by four different stimulus intensities (-4.0 to 1.0 log cd s/m^2^) from *Rax* iCKO and control mice are presented. (D, E) The scotopic amplitudes of a- (D) and b-waves (E) are shown as a function of the stimulus intensity. (F, G) The scotopic implicit time of a- (F) and b-waves (G) are shown as a function of the stimulus intensity. Data are presented as mean ± SD. n = 3 per each genotype. ns, not significant (two-way repeated-measures ANOVA). (H) Representative photopic ERGs elicited by four different stimulus intensities (-0.5 to 1.0 log cd s/m^2^) from *Rax* iCKO and control mice are presented. (I, J) The photopic amplitudes of a- (I) and b-waves (J) are shown as a function of the stimulus intensity. (K, L) The scotopic implicit time of a- (K) and b-waves (L) are shown as a function of the stimulus intensity. Data are presented as mean ± SD. n = 3 per each genotype. ns, not significant (two-way repeated-measures ANOVA).

**(M)** Schedule for tamoxifen injection and harvest of mice. Mice were injected with tamoxifen at P9 or P12 and harvested at 1M (P9→1M or P12→1M).

**(N)** Immunohistochemical analysis of retinas from *Rax* iCKO and control mice (P9→1M or P12→1M). Sections were immunostained using the anti-GFAP antibody. Nuclei were stained with DAPI (blue).

ONL, outer nuclear layer; INL, inner nuclear layer; GCL, ganglion cell layer.
